# Navigating Criminal Governance in Colombia: A Life Stories Approach

**DOI:** 10.1007/s10612-025-09829-0

**Published:** 2025-06-09

**Authors:** Patrick Naef

**Affiliations:** Department of Geography and Environment, University of Geneva, Geneva, Switzerland

## Abstract

This article examines how individuals navigate life in criminalized territories. Drawing on ethnographic research in Medellin, Colombia’s second-largest city, it explores the trajectories of three young men whose lives are deeply intertwined with the criminal elements controlling their neighborhood. Focusing on their ‘life stories’—the significant events and experiences influencing their paths—and their relationships with illegal actors, this anthropological work provides a nuanced understanding of how criminal organizations wield power and maintain governance. Complementing the prevailing approaches to what is generally referred to as ‘criminal governance’, which is often based on quantitative and second-hand data, it shows that this phenomenon is driven not only by rational and predatory dynamics, but also by intimacy and reciprocity. Building on these insights, it proposes the concept of *criminal hegemony* to understand, from a ground-up perspective, how criminal governance is continuously (re)shaped and negotiated.

In Medellin’s downtown area, as well as in the hilly self-settlements surrounding the second city of Colombia, criminal groups colloquially labelled as *combos* ensure some degree of order in the public and private sphere, intervening in petty crime, neighbour-level conflicts or domestic violence. They also provide ‘protection’ in exchange for the infamous *vacuna—*literally the ‘vaccine’, the term used throughout Colombia to describe extortion.

In the past decade, scholars have conceptualized as ‘criminal governance’ the regulation of social spaces, and the control of illegal and legal markets by criminal organizations (Arias 2017; Feldmann and Luna 2022; Lessing 2022; Naef 2023). This authority may be exercised by small groups like street gangs, or by larger structures such as drug cartels and prison gangs (Lessing 2022). Studies on criminal governance have been carried out mainly in the fields of law, economy and political sciences, with a large body of work focusing on the economic impact of extortion. Recently however, scholars in social sciences have provided more qualitative studies that complement pre-existing work, which is often based on quantitative and second-hand data. In contrast to mainstream criminology, which conceives criminal organizations as driven primarily by rational and predatory logic, they have started to take into account ‘the ambiguity and “messiness” of criminal governance’ (Rodgers 2022a, b). Some of them have pointed out its hybrid nature, implying an intertwining of illegal and legal economies, and the co-governance of state and non-state actors (Arias and Rodrigues 2006; Arias and Barnes 2017; Jaffe 2013; Feltran 2020). Moreover, inspired by the seminal work of anthropologists and sociologists who considered violence through the lens of everyday life (e.g., Bourgois 2002; Das 2006; Scheper-Hughes 1993; Taussig 2005; Venkatesh 2002), recent scholarship has shed light on the intimate relations and interactions at play in criminal activities, such as drug trafficking and extortion (Ayuero 2015; Contreras 2012; Jensen and Rodgers 2021; Michelutti 2019; Penglase 2014; Zubillaga et al. 2015). They view the social organization of criminals as ambivalent: rational, entangled with the state, as well as shaped by domestic and intimate factors. They maintain that the aspirations of criminal actors are not driven solely by a cost–benefit logic: their organization is often haphazard, and conflicts are frequently negotiated within the intimate spheres of families, neighbours or long-term acquaintances.

Drawing on these insights, my research offers a more nuanced approach to criminal governance by scrutinising the interpersonal relations and interactions between residents and criminal groups. I explore the life journeys of people living in criminalized territories, and look at the multiplicity and recurrence of these everyday experiences. Criminal governance is shaped by systems of reciprocities that are constantly evolving. In this respect, paying close attention to the lives of people navigating amid crime shows how interpersonal relations are fluid and morph when contexts change, how privileges and obligations at the heart of these disputed spaces may vary from day to day. An individual can be in the good graces of the criminal gang controlling his neighbourhood at one point and threatened by the same group later on. Likewise, a relative who offers protection to a family may also generate insecurity due to his involvement in a gang, or become himself abusive (Deckard and Auyero 2022). Being the brother of a criminal leader can provide you with protection one day and turn into a source of danger the next day.

Digging into the lives of individuals close to criminals also serves to call into question homogeneous social categories such as *victims* and *perpetrators.* Those boundaries are fuzzy and permeable: people regularly step in and out of the world of crime and, more often than not, victimizers have been themselves victims of violence. The ambiguous links individuals maintain with criminal organizations prompted scholars to question the rigid divide between ‘gang members’ and ‘civilians’ (Jackson, Weigand and Tindall 2023). As Dennis Rodgers (2016) showed in Nicaragua, people often collaborate occasionally and irregularly with gangs. Residents can sporadically sell drugs or hide weapons to make ends meet, without being affiliated to a structure. Besides, individuals participating in gang activities are often well integrated in their community. They were born in the territory, they went to school with their neighbours, they have shared meals and beers with other residents, and participated in local soccer tournaments together. Mainstream accounts of gang violence, however, still tend to overlook the permeability of these categories and, as Caylin Louis Moore and Forrest Stuart (2022: 303) have emphasized, such misinterpretations may be detrimental to policy-making:

Prevailing definitions of what counts as a gang, as well as who counts as a gang member, ultimately structure government policies, criminal justice practices, legal decisions, and the broader public discourse with immense consequences for gang-affiliated individuals and their communities.

In sum, many actors involved in crime live diverse and colourful existences. In the course of their life, they can be a brother, a father, a friend, a widower, a victim of forced displacement, a soldier, a member of a gang, an assassin, a convict. An individual who was affiliated to a gang can repent and draw on his experience to participate in youth integration programmes. Inversely, former members of public forces may use their expertise in warfare to join a drug cartel. Cases in Mexico, Colombia and Haiti offer many examples of police officers and regular soldiers reorienting themselves into the criminal underworld, hence exposing the murky waters in which dichotomies such as *civilian/criminal* are conceived.

## Studying Life Stories in Criminalized Contexts

To shed light on these fragmented and entangled identities, I draw on a body of work that has gained momentum in recent decades. This interdisciplinary approach brings together scholars from fields such as sociology, demography, social psychology, and history, in a methodological framework broadly referred to as ‘biographical.’ (Heckhausen and Buchmann 2018; Levy and Bühlmann 2016; Spini et al. 2017; Shanahan, Mortimer and Kirkpatrick 2016; Hollstein 2021; Elder 1974). Among these methods, researchers in social sciences increasingly turned to ‘life stories’ as a tool for gathering ethnographic material and gaining a deeper understanding of human experiences (Peacock & Holland 1993; López-Montero et al. 2022). By focusing on individual trajectories, the aim is not only to explore personal narratives but also to reveal the underlying workings of sociocultural systems.

In biographical methods, life trajectories are seen as movements through social space, and framed as sequences of profiles defined by the roles, positions, events and forms of participation individuals enact in the course of their life. The dynamic interplay between social structures and individual agency is explored, highlighting the opportunities and constraints generated by social institutions and norms, and individual developmental processes (Levi and Bühlmann 2016; Moen et al. 1992; Mitchell 2003; Giele and Elder 1998). The interface between structure and agency is represented by Jutta Heckhausen and Marlis Buchmann as a form of ‘canalization’, ‘where societally and developmentally structured paths guide individuals’ life courses along timed and sequenced paths (2018: 1)’. As they emphasize, individuals navigate life amid opportunities and constraints set in historical contexts, social institutions and the social structure. Their agency enables them to make decisions about the goals they want to achieve and the values attached to these goals (Heckhausen and Buchmann 2018). At the same time, people act in social contexts that have a spatialized and historical structure, where individuals occupy positions reflecting their ambitions and opportunities. By articulating structural landscapes with the individual trajectories traversing them, biographical approaches help to uncover the structured paths in which these journeys take place (Levy and Bühlmann 2016: 30).

The biographical elements presented below describe landscapes and trajectories where positions, roles, constraints, and opportunities are profoundly shaped by illegal armed actors. So far, a large corpus of work in sociology, anthropology, and criminology, has focused on the biographies of criminals, looking primarily at the development of criminal practices from childhood to the present, and at the effects of critical life events on their itineraries (Blokland and Nieuwbeerta 2010; Farrington 2010; Kempf 1987; Sampson and Laub 1993). Scholarship on gangs has driven methodological advancements by strengthening biographical approaches to the study of crime, including longitudinal studies, life-course perspectives, and personal network research designs (PNDRs) (e.g. Rodgers 2016; Roman & Nguyen 2025; Pyrooz et al. 2024). Scholars have examined specific domains like drug trafficking (Li 2024; Jensen and Rodgers 2021), generational conflicts in gangs (Rodger 2022a; Contreras 2012), gender and sexual orientation (Panfil 2017; Carvajal Sanchez 2017; Damme 2024; Baird 2018), and social mobility and entrepreneurship (Rodgers 2022a, b). Rodgers’s (2016, 2022a, b) longitudinal ethnographic studies of gang members in the fictional Nicaraguan neighbourhood of Barrio Luis Fanor Hernández provide the most extensive work in this regard. As he puts it, detailed narratives of gang members’ life trajectories enable researchers to understand their inspirations and incentives in a way that few other methodological approaches have achieved. It also provides more nuanced and humanizing accounts of a population often stereotyped in alarmist and oversimplified representations of violent alterities. In keeping with the vision described above, in which life trajectories are considered to be a sequence of socially defined profiles over time, Rodgers points out ‘vital conjunctures’ or ‘turning points’, when individuals’ lives are impacted by broader structures and processes, or when new opportunities arise. It certainly resonates with visions expressed in the paradigm presented above, where critical biographical events are conceptualized as accumulative changes in participation, positions and roles (Levy and Bühlmann 2016).

## Contested Lives in the Barrios

If paying close attention to the lives of criminals is undoubtedly crucial to get a comprehensive picture of the social organization of crime-ridden neighbourhoods, considering the practices of people who regularly interact with them also provides pertinent insights. It allows to emphasize the agency of individuals navigating amid criminal governance, and to go beyond simplistic visions portraying them as mere passive recipients under the control of dominant actors. It is indeed critical to unpack the strategies, tactics, negotiations and resistances they develop to optimize their security and, broadly, to challenge criminal governance. Drawing on Michel De Certeau’s (1990) theoretical insights, Ben Penglase (2014) has for instance provided a vivid description of everyday life in a favela of Rio de Janeiro, revealing some of the social tactics residents use to ensure their security. By following the trajectories of families living in the urban margins of Brazil, Penglase shows clearly how they navigate these contested spaces by maintaining close relations with drug traffickers, whom he labels ‘dangerous intimates’.

During a decade of research in Medellin, I have conducted repeated interviews with specific actors whom I consider as *in-betweens,* or *brokers*—neighbourhood leaders, relatives of gang members, non-profit collaborators involved in gang mediation—individuals who interact regularly with criminal actors and thus contribute to shaping the criminal governance of their territory. I paid close attention to their most significant life experiences, as well as their relationships and interactions with criminal actors. I suggest that the ties they develop with criminal elements (through kin, friendship, neighbourly relations) critically determine how they navigate life in these disputed spaces. In this relational context, using ‘life stories’ to explore criminal governance—focusing on key events, personal experiences, and interactions within an individual’s biography—helps reveal the often-hidden aspects of the structural landscape. This approach also sheds light on the individual agencies that shape the life paths of those residing in criminalized territories.

The anecdote I present below offers a glimpse of how some mundane events, and the roles associated with someone’s life, can have critical impacts on the social organization. In a neighbourhood of Medellin that I used to visit regularly in the course of my fieldwork, I wondered why a hairdresser did not pay any extortion money to the gang in charge, unlike most of the other businesses. But as I learned more about her personal history, I discovered that her mother was a teacher in an elementary school. Several youngsters who had been in her class and who went on to join the structure currently controlling the barrio remained very fond of their former teacher. Because of this, they always acted benevolently towards her daughter, and spared her the *vacuna.* At first glance, this story may appear trivial. Yet, like many other interactions in the barrios of Medellin, it is significant, since it offers some insights into how criminal groups govern and how residents experience violence and illegality. In the system of reciprocity in which this hairdresser evolves, past actions and events can determine future decisions of those in charge. As this anecdote illustrates, the interactions, relationships, events, and roles that shape the trajectories of individuals living in close proximity to criminal elements are key for understanding the complex social organization of these conflicted spaces.

In line with this example, the next section focuses on the life stories of three young men living in Medellin’s *barrios populares*: the brother of a gang-boss; the son of a leader of one of the main criminal organizations of the city; and finally, a human rights defender who regularly negotiates with the gang controlling his neighbourhood.^1^ These interviews began with a general presentation of their life trajectories, where I asked my interlocutors to highlight events they deemed significant in their biographical journeys. I was particularly interested in the conflicts they had been involved in, the threats they may have faced, and the broader circumstances that contributed to their victimization. We then dwelled on their relationships within the community, with a focus on kinship and friendships, to better understand the role of social networks in managing their everyday security. By doing so, I aimed to uncover some of the tactics they employed to navigate criminalized spaces and, more specifically, the significance of their relationships and interactions with criminal actors.

Conducting fieldwork in these fragile settings, which are marked by significant power dynamics and profound inequalities, calls for a high degree of reflexivity. Furthermore, employing life stories to analyse these contested spaces requires engaging with recent debates on qualitative research that question the efficacy of interviews in capturing human practices and behaviours (e.g. Jerolmack & Khan 2014; Silverman 2013; Hammersley & Atkinson 2007: Miller & Glassner 1997). Factors such as memory biases, personal interests, pressures, and even limitations in language, introduce discrepancies, challenging the researcher’s ability to comprehend human behaviours and accurately describe complex social systems. To address these limitations, methodological pluralism offers a pathway to gain a more comprehensive understanding of these contexts. When it was possible, I have complemented interviews with participant observation, facilitated through collaboration with non-profits dedicated to defending human rights and combating gang recruitment. By observing everyday practices, engaging in daily activities with residents, and participating in their informal conversations, I have been able to develop a more nuanced understanding of the narratives captured in my interviews. This approach proved particularly valuable when exploring topics related to criminal governance, where formal settings and the presence of a voice recorder often introduced a significant filter. By triangulating interview data with insights derived from my observations, I was able to mitigate these challenges and enrich my analysis. Therefore, while most of the findings presented below are the result of formal in-depth interviews, additional insights were obtained through informal conversations that followed these interviews. Besides, I drew upon knowledge gained from other ethnographic approaches to contextualize and reflect on some of the observations presented.

### Brotherhood

I interviewed Sam six years after Johnny, his brother, was assassinated. He was much older than Sam and was therefore a father figure for him.^2^ As a wannabe comedian, Sam is very eloquent and he put a lot of fervour into recalling the significant events in his life, giving me a vivid account of his relation with his brother.

Johnny was a gang leader who controlled a *plaza de vicio,* literally a ‘place of vice’, where illegal activities such as drug-trafficking and prostitution are conducted. He was almost killed by left-wing militias at the end of the eighties, and since then felt a strong hatred for the guerilla. After this failed assassination attempt, he enthusiastically joined a paramilitary organization through his connection with a high-ranking uncle. As Sam explained, his goal was to ‘clean the city of communists and *milicianos*’. Sam took a drastically different path and studied social sciences at the university. In contrast to his brother, he was more inclined to follow in the steps of Che Guevara than adhere to the hard-right philosophy commonly associated with paramilitary groups. This contrast was one of many between them, but it did not weaken their brotherhood. As Sam recalled, his brother was always supportive of his projects.

‘He was my *cucho* (affectionate term to describe a father, a grand-father or an old man). He was my reference and there were no lies between us. I always knew he killed and beat people, that he was a ‘man of war’. One day, he questioned me about my future:

‘*Huevón* what are you going to do with your life? You want to be like me? You want to manage a gang? I’ll teach you. But you know, it is just prolonging the war. I had to do it for protection, not only for me but for all our friends*.* I don’t want you to do that, but you’ll be whoever you want to be. Study or go abroad..? I’ll support it. If you want to put yourself in it [in the *combo*], I’ll support that too.’

As our discussion continued, it became clear to me that Sam’s brother could not be portrayed solely as a criminal. While Sam described the violence associated with his brother’s activities, at the same time he emphasized other aspects of Johnny’s life. He would for instance mention his part-time activity as a clothing designer, his involvement in a band of *currulao*,^3^ and his affection for his two kids*.* Violence was nevertheless a constant feature of his family environment.

My brother once hid a couple of guns behind my mother’s dresser. She found them and woke him up with one of the guns pointed at his face. He needed to understand the notion of respect for our home.

Sam considered such violence among family members as part of a learning process. However, the violence associated with Johnny’s activities ended up being a lot more traumatizing for him. He was only a small child when his brother began his criminal career, and some events affecting his family only made sense later in his life. Sam noted for instance that Johnny would never let anyone photograph him.

We only have one picture of my brother! I also remember some conversations that went on in our house, things that I didn’t understand at the time. After several years, I started to understand these dynamics, things that directly affected us as a family. One thing that I didn’t get is why we were always moving. I remember one day when we received some flowers. My brother immediately started packing his things; he jumped in a car and left. These kinds of things happened often. Sometimes when I was a small kid, I would go to bed in one house and wake up in another.

In the early 2010’s, Sam was finishing his academic studies. He was part of one of the theatre companies of his university. Many in the troupe shared leftist viewpoints and were sympathetic to guerilla ideologies. One day, Sam had an argument with one member of the company, a person who was recruiting students for the Revolutionary Armed Forces of Colombia (FARC). This person complained to one of his cousins implicated in the local *combo*, who decided to pay a visit to Sam. ‘The problem was that I was not at home when the *combo* came to our house. And they did not know that I was Johnny’s brother.’ Sam described with a blend of humour and bitterness the fuss this visit triggered:

They started like: ‘Hey what’s up man? We are looking for somebody. He is fooling around with another guy *muy sano* (literally ‘very healthy’, meaning here, reliable). Well... he needs to keep it shut if he doesn’t want us to break his leg.’

My brother went crazy! Besides, he already knew about the issue, as I had told him the story already. ‘Who the fuck are you to come in my house and ask for my little brother?! And why are you defending a fucking guerreiro?’

After Sam came home, the brothers had a violent dispute. Johnny was furious and wanted to kill the people who had intruded into his house. Sam told him he would denounce him if he did so.

All my life I have kept things separate. I never wanted to take advantage of being the brother of a gang leader*.* It brought me more problems than benefits, so the further away I was, the better.

Sam ended this anecdote by stressing how, in this case, intimate relations had bypassed political ideologies.

It is just crazy that because of family they sent them to scare me. To stop me from preventing recruitment for the FARCs. Ideologically it is absurd! This made my brother even more angry: threatening his little brother on behalf of a guerriero?!

Before this event, Johnny had decided to quit his criminal activities. He wanted to focus instead on his music projects. He played again with his old band and started to offer free classes in community centres. Tragically, this came to an abrupt end: Johnny was shot in the head and killed in 2016, a few days before Easter. The reasons for his killing remain murky, but according to Sam, Johnny had gone into hiding because he was being threatened. The family suspects he was eventually betrayed by a distant cousin living in the same barrio.

As these episodes in his life show, Johnny’s family had an important influence on the road he took, from the beginning of his career when he was introduced to warfare by an uncle, until his death after a cousin supposedly betrayed him. His life also illustrates how criminal actors participate in multiple social fields. Johnny was involved in criminal activities, but he was also engaged in legal and benevolent practices. When he left the world of crime, he could rely on his ‘violence expertise’ (Collins 2008) to help his brother get out of trouble. He drew on the respect he gained during his life to reach a favourable settlement in a dispute with junior gang members.

Finally, this tale of two brothers reveals the challenges of staying close to criminal loved ones without being absorbed into their world. Both brothers maintained tight bonds throughout their lives. Yet, when Sam describes how hard it was to keep his distance from his brother’s activities, he reveals some of the obligations and dangers inherent in belonging to a family involved in crime. After our interview, pointing to his joint, he added that everybody in Medellin contributed to the narco-trafficking: ‘Not only with the weed we smoke, but with the eggs or the cheese we eat, because *combos* control everything. Indirectly, my cousin, my brother, my mom, my father, we are all part of the structure.’

### The Son of the Boss

Livingston is the son of Don Hector,^4^ a leader of one of the main criminal organizations of the city who is now serving a prison sentence. In a series of interviews and discussions we talked about his family context, his aspirations in life and his role in his community. He was very evasive when we talked about his relatives, and it was only after several encounters that he revealed his implication in drug trafficking. He never gave me a clear account of his involvement in his father’s organization.

Although he is in prison, his father is still very influential in neighbourhood affairs. He is well-known in the barrio, and I met various residents who spoke of him in flattering terms, highlighting his sophistication and courtesy. His son, however, had mixed feelings. Livingston often talked about the constraints caused by his family situation. To avoid his father’s enemies, for instance, he and his siblings had to keep a very low profile:

‘Since we were kids, he taught us not to leave the barrio. Now I am less worried about wandering further in the city, although I am still afraid of his enemies. But very few people know that I am his son. […] My father is very strange. Somebody very difficult to understand. Now I don’t blame him, but before he made me very angry. We fought a lot. He shot me twice… My feet… I mean, he didn’t hit me, but he made me dance *joropo*.^5^’

Other constraints concerned Livingston’s professional choices. He had wanted to be a firefighter, but Don Hector would never agree to that. Besides, he was himself extremely pessimistic about his father’s future:

‘My father is a devoted *sicario*^6^… he is dedicated to killing… That is the only thing he has done in his life and he is not going to change overnight. […] He has already served a large part of his sentence, but he will come out worse. Prison made him tougher. It made him more powerful… He is ranked higher in the organisation. He is not only in charge of our barrio; he now rules other territories.'

When I asked about the rest of his family, he added:

‘They are professionals *de manejar la vuelta* (in gang management). Imagine that once someone threw two bombs into my grandma’s house! They blew up everything in the house including the dogs. Well... My family was large, with many men. When the gangsters killed the eldest son of my grandma, the others [his brothers] formed a *combo* and killed those who did not like them.’

‘So, it’s a real family combo?’ I asked.

‘Yeah! Totally family! We were uncles, cousins, nephews... We were all together, and little by little, some were killed... Others were caught.’

Livingston has ten siblings, and as he explained to me, both of his parents came from families involved in criminal activities. Their union thus helped consolidate the stronghold of their *combo*.

My family [on my mother’s side] was in trouble, they had been [forcedly] displaced in the barrio several times. That’s when my parents met. And my *papa,* he was already well-placed in the *combo.* So, he solved her family’s problems and calmed things down.

He was also proud of how his grandparents had helped build his neighbourhood, when they settled in Medellin with nothing. And while he was ambivalent about his relation with Don Hector, he would proudly recount past wars in which his father had fought and gained notoriety. Livingston also portrayed his father as a community-man. On several occasions, he described some of his good deeds for the community, like helping people financially and organizing events for the children during Halloween. When he did so, he criticized the *combo* now in charge who in his opinion had lost any desire to help their community.

Before they were very serious people. Very professional. But after they all got caught, it got kind of lost. Now, yes, they offer security, but nothing social.

He added that now that his father was in jail, some residents would come to him for help or advice. This was confirmed during some of the strolls I took with him in the barrio, when I noticed how he would occasionally help to police the area. Several times, I saw him lecturing a resident who was not following the rules. For instance, he reprimanded young kids who were taking drugs in a place where consumption was forbidden by the gang. He also had a fierce argument with a community leader who brought visitors to the neighbourhood without informing the *combo*. For Livingston, offences in his barrio were always committed by outsiders, by those he qualified as *pirañas*: thieves and criminals from other parts of the city. Such views are far from uncommon in his neighbourhood. It resonates with Don Hector’s orders to his family to stay close to home. The barrio is considered a pure space that risks being contaminated by foreign elements. This standpoint legitimizes the presence of the *combo*, as a body protecting residents from external threats.

During one of our last encounters, I asked Livingston if people involved in the local *combo* carried out certain rituals or had particular ways of paying tribute to the dead. He dismissed the idea out of hand, stressing that here, when people died it was for a reason: ‘All those who died, it was because they were *torcidos* (crooked)… Or because it had to be’. Once again, such comments shed light on Livingston’s ambivalent vision of the *combo* and his own family*.* While members of criminal organizations may deserve their fate, he nevertheless often acknowledges that they are also legitimate actors providing social order. As his relation with Don Hector exemplifies, and as Penglase commented in urban Brazil, they are dangerous intimates, inspiring fear and anger, but also respect and pride.

The fragments of Livingston’s life that I have pieced together offer a view of some of the obligations one faces when being part of the family of a criminal leader in Medellin. It shows the importance of keeping a low profile, and limiting one’s movements and professional choices. It also reveals the responsibilities one must take on when critical events occur, as the incarceration of Don Hector illustrates. When his father was jailed, Livingston, willingly or not, took on responsibilities in the community, acquiring new privileges, but also new obligations. In the words of Levy and Bühlmann (2016), he endorsed a role, with the rights, duties, interests and conflicts related to it. Finally, Livingston’s family history confirms that kinship may be key to the formation of criminal groups in Medellin. In a context where people are often displaced and where structures of power can change from one day to the next, the family can offer some stability. When one’s relatives are deeply involved in crime, however, it can also be a breeding ground for uncertainty and danger.

### Family Resistance

I met Kanabico when he was a little over 30 years old.^7^ He is well-known in Medellin’s activism spheres and used to discussions with journalists and academics. His mother was a fervent community worker and Kanabico followed in her footsteps, becoming a youth activist. As a result, the family has been at odds with the criminal actors ruling their barrio. The organization in charge dates from the eighties. It went from being a gang affiliated to Medellin’s Cartel to becoming a major organization operating in several districts of the city. Kanabico explained that although the structure itself has been stable for the last two decades, local gang leaders change regularly. Hence, every shift in power generates new tensions: previously settled conflicts re-surface and the family needs to negotiate its security again. What Kanabico describes as the ‘first shock with the *combo*’ occurred in 2002. His mother was a leader of a local parents’ association and she intervened to prevent the misappropriation of food meant for schoolchildren.

‘I always had a relation [based on] shocks with my family and paramilitary *combos*. […] It started with a graffiti on our house signed *AUC*^8^: “Guerrillero, put on camouflage or die in civilian clothes”. We erased it and went on. [But] things were clear.'

‘Later, a group went to our house with a letter identifying my mother as a military objective, giving us 24 h to leave. Surprisingly, one of the guys was a friend of my brother. They had studied together their whole life. It went on like that:'

‘Oh! How come? She’s your mum, she’s your *cucha*! No, brother, let’s talk. Tell her that we’re not going to do anything, but tell her to stay away from school stuff.’

‘This was the first time, but after that, every year or so… Every time there is a new leader, my mother is threatened again. As if they had a profile [page] on her at the Oficina de Envigado.'^9^

Although the family received many threats, some of his mother’s relations meant they could navigate in relative security. For instance, she had worked in a childcare centre more than a decade ago, so she could count on the support of gang members that she had looked after in this school. As Kanabico emphasized, the tensions they experienced varied widely, depending on the actors involved. Nevertheless, they lived in constant fear of threats. Despite it all, Kanabico made it clear that they had no intention of leaving their barrio. ‘My mother has forty years of community work behind her. She has always said that she owes nothing to anybody and will not leave.’ He also described the intrafamily wars that occurred when some of his relatives were fighting in opposite sides*:*

One from the *bloque* Metro, the other from the [*bloque*] Nutibara*,* both died because of these conflicts... brothers in opposing *combos*. I never quite understood the conflict between Metro and Nutibara […] It was always a shitshow. It sounds cruel to say it like that, but their death was a relief for the family.^10^

On another occasion, however, his family ties within the *combo* gave him leverage to resolve a dispute. One day, for example, Kanabico found himself in trouble after speaking out on television about recent threats. An acquaintance of his deceased cousins intervened on his behalf, successfully defusing the situation. As Kanabico put it, these conflicting dynamics exemplify the paradoxes inherent in what he termed the *familia antioqueña.*^11^ In the same family, victims of threats interact with relatives closely connected to gangs. At times, these connections can be beneficial, while at other times this dangerous intimacy becomes a significant source of tension.

Kanabico is involved in anti-militarist activism and, as his nickname suggests, is a staunch advocate of the legalization of cannabis. Consequently, his political activities have led to conflicts not only with criminal groups but also with the authorities. He described, for instance, a recent death threat he and his mother received. The *combo* accused them of being *sapos* (snitches), insinuating that they had reported a shipment of drugs and weapons to the police. However, through their connections, Kanabico discovered that a police officer had set them up:

The police made up a story that we had called from my house to report a shipment. If there is one thing we have learned, it is that first, we don’t call the police from our own telephones, and not even from our homes. Two, we don’t call the police at all!

Kanabico also thought that the family’s involvement in politics and human rights defence provided them with some protection. He recalled an interaction he had with a gang member*.*

He looked at me and said: ‘*Venga*! What is it that you are doing? I see you have a lot of support. Many people came to intervene in your favour.’

‘I worked for the community. I do politics.’

‘He told me: “No… You and your mother are really protected. There are a lot of people interested in protecting you…”.’

Kanabico added that this remark was still unclear for him, but he believed that his involvement with certain non-profits, while often putting him at risk, might also offer some degree of protection: ‘It seemed that various human rights organizations were mentioning us as people to be protected. This acted as an alert for the *combo*.’ He nevertheless acknowledged that this relative protection was less effective outside the confines of his barrio. During the Covid-19 pandemic, for instance, he publicly denounced the theft of municipal food aid, directly accusing a gang from a neighboring area.

It became very tense because it was a complaint against another *combo*, against a guy who had nothing to do with me. For a moment, I was like ‘Shit, I fucked up’. I should not have spoken so bluntly. But when this *combo* started to ask for me, the others said: ‘don’t mess with this guy, he is protected.’

Since 2002, Kanabico and his mother have maintained a tense relationship with the criminal elements controlling their barrio. Over the course of two decades—marked by threats, the poisoning of their dogs, and the vandalism of their home—they have built significant social networks, drawing on their family ties and long-standing relationships. They knew many of the old *combo* members, and some of their relatives were even involved in criminal activities. Kanabico stressed that their many conversations and negotiations with criminal actors fostered a process of resistance. ‘We showed them that we were not afraid. Of course, it helps that it has always been present in the family… in the house. I mean… It does not make sense to be afraid of guys we studied with.’ When we met, Kanabico felt relatively secure within the boundaries of his neighbourhood. His remarks suggest that his social capital play a significant role in this sense of safety; he mentioned that he was currently regarded as someone ‘to protect’. However, his life story also highlights the precarious nature of this situation: at times, protected by criminals, public authorities, or civil society organizations, and at other times, threatened by the very same forces.

## Reciprocity Within Criminal Hegemony

The concept of reciprocity has emerged as a cornerstone of anthropological study and a pivotal theme in the social sciences across the years (e.g. Mauss 2002; Malinowski 1922; Sahlins 1972). In his founding work—*Essai sur le don* (1925)—Marcel Mauss argued that exchanges extended beyond immediate transactions, generating long-term obligations that critically shape social structures. ‘Gifts’, he proposed, carry an inherent expectation of reciprocity, binding individuals, maintaining social cohesion, and balancing power dynamics. Thus, giving, receiving and reciprocating are not solely economic acts, but have deeply rooted social and moral dimensions. Moreover, these mechanisms are not fixed: reciprocity is continuously (re)negotiated and social bonds are constantly (re)formed.

In the wake of this work, scholars have increasingly examined the role of reciprocity in contexts of inequality and violence, focusing on how it is shaped by interpersonal relationships and everyday practices (e.g., Lomnitz 1977; Arias & Rodrigues 2006; Deckard & Auyero 2022; Michelutti 2019). In criminalized territories marked by significant relations of domination, it has long been observed that the authority of criminal actors is not sustained solely through violence and fear. Rather, it is maintained through a combination of coercion, patronage, collaboration and cultural resonance (e.g., Bobbio 1993; Barnes 2017; Doyle 2021; Arias 2017). Marxist philosopher Antonio Gramsci’s concept of hegemony provides a valuable lens for understanding how power operates through a combination of coercion and consent between dominant and subordinate classes (Gramsci 1971). Building on his insights and shifting the focus to non-economic relations between residents and illegal actors, I introduce the concept of **‘**criminal hegemony**.’** This framework highlights how the threat of violence, coupled with the cultivation of consent, enables criminal organizations to maintain their domination while keeping a balance between various social forces. When Livingston describes how his father rules, he highlights the delicate balance between coercion (Don Hector as a ruthless killer) and the pursuit of consent (Don Hector as a caretaker of the barrio). In Gramscian terms, Don Hector, as a member of the hegemonic class, imposes order through violence in his barrio and family while also addressing the interests of those over whom he exercises hegemony. As suggested by the three biographical accounts, this *criminal hegemony* is established through daily practices, beliefs, and norms.

Indeed, following Gramsci’s insight, hegemony is not only enforced through coercion but also through the persuasion of subordinate classes to adopt the values and ideas of the dominant ones. Examples from the life stories of my research participants illustrate that, while they frequently challenge criminal governance, they sometimes align with the vision put forth by those in power. For instance, Livingston repeatedly underscored the importance of protecting the barrio from external threats, a perspective heavily promoted by the *combos* to legitimize their control. Similarly, although Sam has continually fought to distance himself from his brother’s activities, he ultimately acknowledged that everyone participates in illegality by engaging in activities or markets controlled by the *combos*. In this context, shared values and ideas give rise to networks of alliances: social equilibrium is maintained through micro-hierarchies, not only between residents and criminal groups but also within the criminal groups themselves. The life stories disclosed by Sam and Kanabico vividly illustrate the conflicts among criminal elements and the role of personal histories (and shared values) in shaping legitimacy. As an example, although Johnny was ultimately killed, he frequently leveraged his status as a respected gang leader to further his own interests.

Gramsci’s concept of hegemony thus provides a compelling framework for analysing how criminal governance is (re)shaped and negotiated. It allows us to understand how everyday practices, cultural representations, interpersonal relationships, and social interactions contribute to reproduce inequalities and hierarchies. At the same time, it demonstrates how these dynamics are leveraged by individuals living under the rule of criminal groups to resist and challenge their subordination. Residents of criminalized territories, while adapting to fluctuating situations, actively seek opportunities to foster counter-hegemonic forces. This duality is particularly evident in the actions of Kanabico and his mother, who must continually adapt to shifting criminal orders yet utilize their social networks effectively to resist the domination of the *combos*. Their trajectories not only highlight their agency in navigating—and resisting—these pressures but also reveal how broader structural changes, such as shifts in organizational leadership within their barrio, profoundly shape their lived experiences. Rooted in reciprocal systems that are constantly evolving, criminal hegemony is far from static. It is a negotiated and disputed process, where dominant groups must continuously strive to assert and maintain their leadership. Life stories serve as a compelling means for capturing these dynamic contexts, shedding light on the evolving nature of violent orders and the strategies employed by communities to navigate and respond to criminal governance.

Additionally, glimpses into the lives of Sam and Livingston show how their intimate ties to criminal elements can constrain their ability to shape their life paths. While Sam feels deep respect and affection for his brother, he constantly struggles to distance himself from the world of crime. Similarly, although Livingstone repeatedly expressed his desire to remain far from his father’s organization, Don Hector’s incarceration reinforced his status as a ruler within their barrio. Ultimately, Johnny’s attempts to give up his criminal activities were thwarted—first when he had to help Sam when he was in a difficult situation, and later when betrayal led to his tragic death. Echoing Heckhausen and Buchmann (2018), these examples illustrate how individuals are sometimes ‘locked in’ or ‘canalized’ by critical societal conditions. They correspond to Li’s (2024) vision of social structures as both the medium and outcome of social action. In his examination of former heroin drug dealers’ life trajectories in China, he asserts that while their lives are influenced by social structures, their criminal trajectories also contribute to reproducing these structures.

Finally, as noted earlier, addressing criminal governance through the testimonies of individuals connected to criminal elements raises questions about how accurately these narratives reflect behaviours and interactions over recent decades. Some accounts are second-hand—for instance, when Sam or Livingston recount the experiences of their relatives—and may be influenced by interpretation or memory bias. At times, these biographical extracts can be corroborated by other narratives. For example, as Don Hector was well-known in his barrio, I was able to gather additional accounts about him and his family. However, due to their inherently personal nature, this type of information is often difficult to verify comprehensively. Nevertheless, this emic approach to criminal governance offers profound insights into the lived experiences of individuals navigating daily life in criminalized territories. Therefore, while I acknowledge the limitations of conducting interviews—particularly when they involve life stories—I remain convinced that they are a powerful tool for understanding social spaces. Exploring the key events, experiences and relationships that shaped the lives of these individuals allowed me to develop a fine-grained understanding of how they responded to criminal governance, how these interactions influenced their decisions and their position in the social fabric and, broadly, how vulnerability and security, collaboration and resistance, were strengthened or undermined in the margins of Medellin.

## Conclusion

These accounts portray three individuals, young adult males, who use their personal networks in various ways, spanning adaptation, collaboration and resistance. Sam and Livingston both shared family ties with criminal leaders, and their situations confirmed that such intimacy was not exempt from risks. If they undoubtedly enjoyed some privileges by being close to dangerous intimates, they were nonetheless repeatedly exposed to violence. Their relations appeared toxic when they were limited in their professional aspirations, and constrained when trying to stay away from criminal activities, or being at risk of receiving a stray bullet. Yet, both of them expressed respect and pride for their relatives. Moreover, the violence was generally seen as an external factor, while their relations were rarely considered abusive. In contrast to Livingston and Sam, the family ties Kanabico shared with criminal elements were weaker, and his interactions with criminal groups were sometimes a source of insecurity. On others occasions, however, he was able to use his personal network to his own advantage, and could receive some degree of protection from various actors. Ultimately, although he and his mother live under constant threat, their social network has provided them with a certain degree of security.

As these life stories demonstrate, social networks are crucial for communities navigating violent contexts. Yet, some of the extracts presented above bring insights that call into question romanticized visions of *social capital* and *communities* as being inherently positive. In violent environments, they can certainly help foster security, but they can also cause violence, incertitude and insecurity. In this respect, the lives of city dwellers navigating at the margins need to be examined from a dynamic perspective, and the reciprocal effects of other trajectories should be thoroughly scrutinized. As Dario Spini, Laura Bernardi and Michel Oris emphasize, vulnerability and resilience processes are shaped with time and spillovers between life domains, ‘producing positive or negative effects on multiple trajectories at the same time (Spini et al. 2017: 9)’. In the case of Kanabico, for instance, interdependent trajectories such as family and activism are deeply entangled, sometimes contributing to his security and at other times generating risk.

Other examples in these extracts call for caution when dealing with projects and discourses using binary conceptions like *gang/community* or *crime/legality*. As some comments reveal, having a leading position in a criminal organization is a role among others in someone’s life story. Other forms of participation may include the practice of an art, a part-time legal job, or the provision of community work. Participations, positions and roles can change, pointing again to the importance of conceptualizing life trajectories in dynamic terms. This means paying close attention to the sequences and vital conjectures that shape the life of an individual, and considering attentively the interaction between social structures and individual agency.

Finally, through these snapshots of lives in the barrios, my objective was to contribute to a more comprehensive vision of how people navigated in criminalized territories. By examining the personal experiences of individuals closely tied to criminal actors, I wished to show the complexity of interacting with dangerous intimates. For scholars working in crime, events taking place within the familial context may appear irrelevant, yet, as suggested earlier, the domestic sphere is crucial for conflict management. Besides, while a large corpus of work has already addressed the ways dominant groups such as political elites and criminal organizations produce (in) security, only limited attention has been paid to the variety of residents’ personal experiences and practices aimed at ensuring their own security. However, ‘residents’—the small business owner, the hairdresser, the schoolteacher—are often on the front line where criminal groups govern. They have limited access to administrative and legal bodies, and intimate spheres are thus key areas for navigating criminal governance. By focusing on their life stories, scholars gain an innovative tool that helps them develop a more refined and nuanced understanding of how violence shapes and structures everyday life.

## Footnotes

1 All interview extracts presented in this article have been translated from Spanish by the author.

2 I interviewed Sam in Medellin in April 2022, and I gave both brothers pseudonyms to preserve Sam’s anonymity.

3 A musical style popular with Afro-descendant communities in Colombia, especially in the Pacific region.

4 I interviewed Livingston in February 2020. I also interacted with him during more informal encounters, where I for instance met several members of his family. I gave him and his father pseudonyms to insure his anonymity.

5 Traditional dance and music in Colombia and Venezuela.

6 Hitman.

7 I interviewed Kanabico in April 2022; he gave his approval to be quoted without being anonymized.

8 The United Self-Defense Forces of Colombia (AUC), a far-right national paramilitary umbrella organization.

9 La Oficina de Envigado is the name given to a network of criminal organizations active in Medellin and broadly in the Valle de Aburrá. It became the ‘Oficina del Valle de Aburrá’ in the midst of turf wars that took place at the beginning of the 2000’s.

10 Nutibara and Metro are the names of two paramilitary factions who fought in Medellin between the late 1990s and early 2000s.

11 Antioquia is the department in which Medellin is situated.

## References

[CR1] Arias, E. D. (2017). Criminal Enterprises and Governance in Latin America and the Caribbean. Cambridge: Cambridge University Press.

[CR2] Arias, E. D., & Barnes, N. (2017). Crime and plural orders in Rio de Janeiro, Brazil. Current Sociology, 65(3), 448–465. 10.1177/0011392116667165

[CR3] Arias, E. D., & Rodrigues, C. D. (2006). The Myth of Personal Security: Criminal Gangs, Dispute Resolution, and Identity in Rio de Janeiro’s Favelas. Latin American Politics and Society, 48(4), 53–81.

[CR4] Baird, A. (2018). Becoming the ‘Baddest’: Masculine Trajectories of Gang Violence in Medellín. Journal of Latin American Studies, 50(1), 183–210. 10.1017/S0022216X17000761

[CR5] Barnes, N. (2017). Criminal Politics: An Integrated Approach to the Study of Organized Crime, Politics, and Violence. *Perspectives on Politics,* 15(4), 967–987.

[CR6] Blokland A., & Nieuwbeerta, P. (2010). Life Course Criminology. In S., G. Shoham, P. Knepper, M. Kett (Eds.) International Handbook of Criminology (51–94). London: Routledge.

[CR7] Bobbio, N. (1993). *Il futuro della democrazia*. Torino : Einaudi.

[CR8] Bourgois, P. (2002, December). In Search of Respect: Selling Crack in El Barrio. Cambridge Core; Cambridge University Press.

[CR9] Carvajal Sanchez, F. (2017). Se faire mâle dans le Medellín des années du Cartel. Sciences et actions sociales, 7.

[CR10] Collins, R. (2008). Violence: A Micro-sociological Theory. Princeton: Princeton University Press.

[CR11] Contreras, R. (2012). The Stickup Kids: Race, Drugs, Violence, and the American Dream. Berkeley: University of California Press.

[CR12] Damme, E. V. (2024, August 5). Jennifer, Honduras: 15 ans et toute première femme cheffe de gang. The Conversation.

[CR13] Das, V. (with Cavell, S.). (2006). Life and Words: Violence and the Descent into the Ordinary. Berkeley: University of California Press.

[CR14] Certeau de, M. (1990). L’invention du quotidien. Paris: Folio Essais.

[CR15] Deckard, F. M., & Auyero, J. (2022). Poor People’s Survival Strategies: Two Decades of Research in the Americas. Annual Review of Sociology 48(1), 373–95.

[CR16] Doyle, C. (2021). “The Criminal Actors Have a Social Base in Their Communities”: Gangs and Service Provision in Medellín, Colombia. *Latin American Politics and Society, 63*(1), 27–47.

[CR17] Elder Jr., G. H. (1974). Children of the Great Depression: Social change in life experience. Chicago: University of Chicago Press.

[CR18] Farrington, D. P. (2010). The Sage Handbook of Criminological Theory. London: Sage Publications.

[CR19] Feldmann, A. E., & Luna, J. P. (2022). Criminal Governance and the Crisis of Contemporary Latin American States. Annual Review of Sociology, 48(1), 441–461. 10.1146/annurev-soc-030420-124931

[CR20] Feltran, G. (2020). The Entangled City: Crime as Urban Fabric in São Paulo. Manchester: Manchester University Press.

[CR21] Giele, J. Z., & Elder Jr., G. H. (Eds.). (1998). Methods of life course research: Qualitative and quantitative approaches. London: Sage Publications.

[CR22] Gramsci, A. (1971). Selections from the prison notebooks (Q. Hoare & G. Nowell Smith, Eds. & Trans.). New York: International Publishers.

[CR23] Hammersley, M., & Atkinson, P. (2007). *Ethnography: Principles in Practice*. London: Routledge.

[CR24] Heckhausen, J., & Buchmann, M. (2018). A Multi-Disciplinary Model of Life-Course Canalization and Agency. Advances in Life Course Research, 41. 10.1016/j.alcr.2018.09.002 10.1016/j.alcr.2018.09.002 36738025

[CR25] Hollstein, B. (2021). Promises and pitfalls of qualitative longitudinal research. 12(1), 7-17. 10.1332/175795920X16040851984946

[CR26] Jackson, A., Weigand, F., & Tindall, T. (2023). Crime and communities: Life under criminal group control. ODI: Think Change. https://odi.org/en/publications/crime-and-communities-life-under-criminal-group-control/

[CR27] Jaffe, R. (2013). The Hybrid State: Crime and Citizenship in Urban Jamaica. American Ethnologist. 10.1111/amet.12051

[CR28] Jensen, S., & Rodgers, D. (2021). The intimacies of drug dealing: Narcotics, kinship and embeddedness in Nicaragua and South Africa. Third World Quarterly. 43(11), 1–19. 10.1080/01436597.2021.1985450

[CR29] Jerolmack, C., & Khan, S. (2014). Talk Is Cheap: Ethnography and the Attitudinal Fallacy. *Sociological Methods & Research,* 43(2), 178–209.

[CR30] Kempf, K. L. (1987). Specialization and the Criminal Career. Criminology, 25(2), 399–420. 10.1111/j.1745-9125.1987.tb00803.x

[CR31] Lessing, B. (2022). Conceptualizing Criminal Governance. Perspectives on Politics, 1–20. 10.1017/S1537592720001243

[CR32] Levy, R., & Bühlmann, F. (2016). Towards a socio-structural framework for life course analysis. Advances in Life Course Research, 30, 30–42. 10.1016/j.alcr.2016.03.005

[CR33] Li, D. (2024). Lost on the Road to Prosperity: Trajectories to Drug Dealing of the Chinese Heroin Generation. Critical Criminology. 10.1007/s10612-024-09796-y

[CR34] Lomnitz, L. A. (1977). *Networks and Marginality: Life in a Mexican Shantytown*. Cambridge, MA: Academic Press.

[CR35] López-Montero, R., García-Navarro, C., Delgado-Baena, A., Vela-Jiménez, R., & Sianes, A. (2022). Life stories: Unraveling the academic configuration of a multifaceted and multidisciplinary field of knowledge. *Frontiers in Psychology, 13*, 960666. 10.3389/fpsyg.2022.960666 36467204 10.3389/fpsyg.2022.960666 PMC9714439

[CR36] Malinowski, B. (1922). *Argonauts of the Western Pacific*. London : Routledge.

[CR37] Mauss, M. (2002) (Original work published 1923). *Essai sur le don*. Paris : Presses Universitaires de France

[CR38] Michelutti, L. (2019). Circuits of Protection and Extortion: Sovereignty in a provincial North Indian town. In D. Gilmartin, P. Price, & A. Engelsen Ruud (Eds.) South Asian Sovereignty (pp. 150–171). Oxfordshire: Routledge

[CR39] Miller, J., & Glassner, B. (1997). The ‘Inside’ and the ‘Outside’: Finding Realities in Interviews. In D. Silverman (Ed.), *Qualitative Research: Theory, Method and Practice* (pp. 125-139). London : Sage.

[CR40] Moen, P., Dempster-McClain, D., & Williams, R. M. (1992). Successful Aging: A Life-Course Perspective on Women’s Multiple Roles and Health. American Journal of Sociology, 97(6), 1612–1638. 10.1086/229941

[CR41] Moore, C. L., & Stuart, F. (2022). Gang Research in the Twenty-First Century. Annual Review of Criminology, 5(1), 299–320. 10.1146/annurev-criminol-030920-094656

[CR42] Naef, P. (2023). The Criminal Governance of Tourism: Extortion and Intimacy in Medellín. Journal of Latin American Studies, 55(2).

[CR43] Panfil, V. R. (2017). The Gang’s All Queer: The Lives of Gay Gang Members. New York: New York University Press.

[CR44] Peacock, J. L., & Holland, D. C. (1993). The Narrated Self: Life Stories in Process. *Ethos, 21*(4), 367–383. 10.1525/eth.1993.21.4.02a00010

[CR45] Penglase, R. B. (2014). Living with Insecurity in a Brazilian Favela: Urban Violence and Daily Life. Rutgers University Press. https://www.jstor.org/stable/j.ctt9qh1fx

[CR46] Pyrooz, D. C., Leverso, J., Sanchez, J. A., & Densley, J. (2024). History, linked lives, timing, and agency: New directions in developmental and life-course perspective on gangs. *Annual Review of Criminology, 7*(1), 105–127. 10.1146/annurev-criminol-022222-

[CR47] Rodgers, D. (2016). Critique of Urban Violence: Bismarckian Transformations in Managua, Nicaragua. Theory, Culture & Society. 33(7), 85-109. 10.1177/0263276416636202

[CR48] Rodgers, D. (2022a). (Il)legal Aspirations: Of Legitimate Crime and Illegitimate Entrepreneurship in Nicaragua. Latin American Politics and Society, 64(4), 48–69. 10.1017/lap.2022.27

[CR49] Rodgers, D. (2022b). ¡A Nosotros, nos Tienen que Respetar! (They Have to Respect us!): Gangs, Inter-Generational Conflict, and Graduated Governance in Urban Nicaragua. Critical Criminology, 30(1), 177–192. 10.1007/s10612-022-09616-1

[CR50] Rodgers, D. (2024). Of Drugs, Tortillas, and Real Estate: On the Tangible and Intangible Benefits of Drug Dealing in Nicaragua. In E. Desmond Arias & T. Grisaffi (Eds.), Cocaine: From Coca Fields to the Streets (pp. 190–208). Durham: Duke University Press.

[CR51] Roman, C. G., & Nguyen, T.-T. (2025). Beyond the collective: Personal networks in the study of street gang processes. *Journal of Criminal Justice, 96*, 102338. 10.1016/j.jcrimjus.2024.102338

[CR52] Sahlins, M. (1972). Stone Age Economics. Piscataway, NJ: Transaction Publishers.

[CR53] Sampson, R. J., & Laub, J. H. (1993). Crime in the Making: Pathways and Turning Points Through Life. Cambridge, MA: Harvard University Press.

[CR54] Scheper-Hughes, N. (1993). Death Without Weeping: The Violence of Everyday Life in Brazil Berkeley: University of California Press.

[CR55] Shanahan, M. J., Mortimer, J. T., & Kirkpatrick Johnson, M. (Eds.). (2016). Handbook of the Life Course: Volume II. Cham: Springer International Publishing. 10.1007/978-3-319-20880-0

[CR56] Spini, D., Bernardi, L., & Oris, M. (2017). Toward a life course framework for studying vulnerability. Research in Human Development, 14(1), 5–25. 10.1080/15427609.2016.1268892

[CR57] Taussig, M. (2005). Law in a Lawless Land: Diary of a Limpieza in Colombia. Chicago: University of Chicago Press. https://press.uchicago.edu/ucp/books/book/chicago/L/bo3680337.html

[CR58] Venkatesh, S. A. (2002). American Project: The Rise and Fall of a Modern Ghetto. Harvard University Press.

[CR59] Zubillaga, V., Llorens, M., & Souto, J. (2015). Chismosas and Alcahuetas: Being the Mother of an Empistolado within the Everyday Armed Violence of a Caracas Barrio. In P. Bourgois & N. Scheper-Hughes (Eds.) Violence at the Urban Margins (pp. 162–188). New York: Oxford University Press. 10.1093/acprof:oso/9780190221447.003.0008

